# Boundary Complexity of (Sub-) Cortical Areas Predict Deep Brain Stimulation Outcomes in Parkinson’s Disease

**DOI:** 10.21203/rs.3.rs-5537857/v1

**Published:** 2024-12-12

**Authors:** Devin Schoen, Skyler Deutsch, Juhi Mehta, Sarah Wang, John Kornak, Philip Starr, Doris Wang, Jill Ostrem, Ian Bledsoe, Melanie Morrison

**Affiliations:** University of California - San Francisco; University of California - San Francisco; University of California - San Francisco; University of California, San Francisco; University of California San Francisco; University of California; University of california; University of California - San Francisco; University of California - San Francisco; University of California - San Francisco

## Abstract

While deep brain stimulation (DBS) remains an effective therapy for Parkinson’s disease (PD), sources of variance in patient outcomes are still not fully understood, underscoring a need for better prognostic criteria. Here we leveraged routinely collected T1-weighted (T1-w) magnetic resonance imaging (MRI) data to derive patient-specific measures of brain structure and evaluate their usefulness in predicting changes in PD medications in response to DBS. Preoperative T1-w MRI data from 231 patients with PD were used to extract regional measures of fractal dimension (FD), sensitive to the structural complexities of cortical and subcortical areas. FD was validated as a biomarker of Parkinson’s disease (PD) progression through comparison of patients with PD and healthy controls (HCs). This analysis revealed significant group differences in FD across nine brain regions which supports its utility as a marker of PD. We evaluated the impact of adding imaging features (FD) to a clinical model that included demographics and clinical parameters—age, sex, total number and location of DBS electrodes, and preoperative motor response to levodopa. This model aimed to explain variance and predict changes in medication following DBS. Regression analysis revealed that inclusion of the FD of distributed brain areas correlated with post-DBS reductions in medication burden, explaining an additional 13.6% of outcome variance (R^2^=0.388) compared to clinical features alone (R^2^=0.252). Hypergraph-based classification learning tasks achieved an area under the receiver operating characteristic curve of 0.64 when predicting with clinical features alone, versus 0.76 when combining clinical and imaging features. These findings demonstrate that PD effects on brain morphology linked to disease progression influence DBS outcomes. The work also highlights FD as a potentially useful imaging biomarker to enhance DBS candidate selection criteria for optimized treatment planning.

## Introduction

Parkinson’s disease (PD) is a neurodegenerative disorder characterized by motor symptoms, including tremor, rigidity, bradykinesia, and postural instability, as well as non-motor symptoms, such as cognitive impairments and mood disturbances^1,2^. PD is marked by a pronounced loss of dopaminergic neurons in the brain, particularly in the substantia nigra^3^. Currently, PD affects millions of individuals worldwide, with higher incidence rates observed in older adults. As life expectancy continues to rise and the population ages, the burden of PD is expected to escalate further^4^.

While there is no cure for PD, various options are available to manage symptoms and improve patients’ quality of life. Medication remains the cornerstone of treatment, aiming to replenish dopamine levels or enhance its effectiveness^5^. However, in cases where medication can no longer control symptoms adequately, an adjunct therapy like deep brain stimulation (DBS) may be considered^6^. DBS is a neuromodulation device-based therapy that involves the surgical implantation of electrodes into targeted brain regions, typically the subthalamic nucleus (STN) and the globus pallidus interna (GPi), to deliver controlled electrical impulses that modulate abnormal neuronal activity and alleviate motor symptoms^7^. As the disease progresses, fluctuations in motor control with medication management alone is often a factor in determining the suitability of deep DBS as a therapy for a patient. DBS is useful in improving motor fluctuations, reducing medication related side effects including dyskinesia, and has been shown to improve patients’ quality of life. Consequently, the use of DBS in PD is on the rise, as is the number of candidates due to the increasing prevalence of PD^8,9^.

Despite its efficacy in alleviating PD motor symptoms in many patients, outcomes following DBS exhibit significant variability, with instances of notable improvement juxtaposed with cases displaying limited response or even worsening of symptoms^10–12^. Current selection criteria attempt to predict these outcomes based on the responsiveness of patients’ motor symptoms to dopaminergic medications^13,14^. However, medication responsiveness has been shown to have limited predictive accuracy^15–18^, presenting the need for more accurate and objective prognostic markers that can reliably identify the most ideal DBS candidates.

Magnetic resonance imaging (MRI) is integral to DBS implementation and thus a convenient tool for extracting quantitative biomarkers and predicting individual outcomes. Advanced MRI techniques probing brain function, tissue microstructure, and iron content via tissue magnetic susceptibility have previously been shown to be related to DBS outcomes^19–25^, though these sequences are not always routinely collected as a part of routine clinical work-up. In contrast, T1-weighted (T1-w) anatomical MRI is universally collected for screening and treatment planning for every DBS patient. Despite its ubiquitous collection, researchers have yet to derive robust biomarkers from T1-w images, though leveraging their widespread availability and routine acquisition could offer valuable insights into individual treatment outcomes that are easily translatable to clinical practice.

Here we focus on extracting regional brain measures of fractal dimension (FD) from T1-w images as potential features to explain variance in PD DBS outcomes. FD quantifies the irregularity of a structure’s surface^26,27^, and in the context of PD and DBS, offers a unique perspective on the underlying structural changes associated with disease progression and treatment response. This morphometric feature has previously been used to distinguish PD from normative brains,^28,29^ as well as to study subtle changes in neurodegeneration^30^.

The first part of this study validates FD as a biomarker of PD progression using open-source, multi-center data. We then leverage our large single-center dataset comprised of preoperative T1-w images from 231 individuals with PD and DBS and employ hypergraph techniques for data representation, variance modeling, and classification learning tasks, to evaluate the potential usefulness of FD as a biomarker for DBS candidate selection.

## Results

### Validation of FD as a PD biomarker.

**Figure 1:**
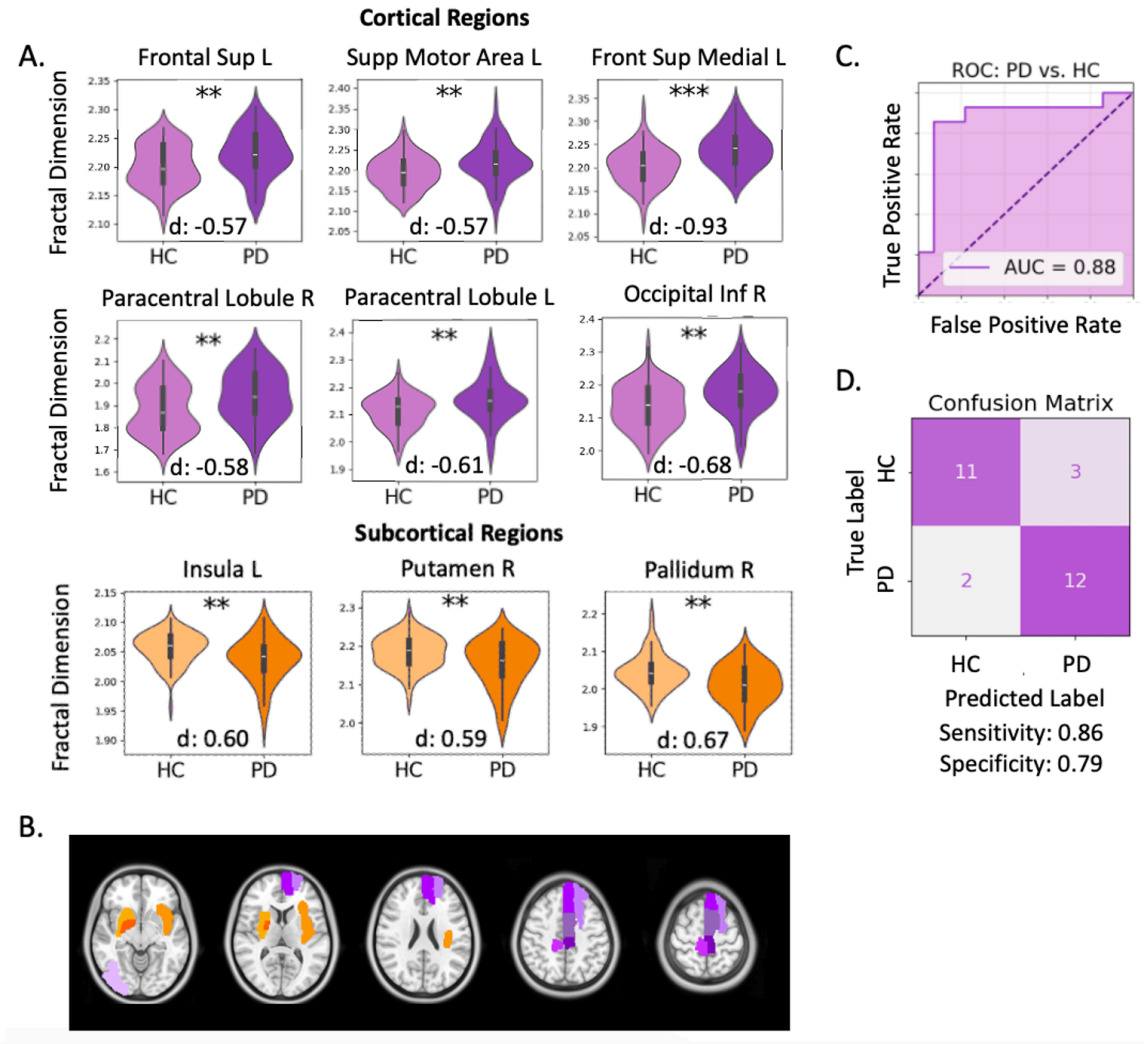
Fractal dimension distinguishes patients with Parkinson’s disease (PD) from healthy controls (HCs): (A) FD of nine brain regions were significantly different for PD (n=70) and HCs (n=70). Asterisks indicate the adjusted significance level of a two-sided t-test (**p<0.01, ***p<0.001) and Cohen’s d effect sizes are presented. (B) Spatial distribution of the 9 regions where FD was found to be significantly different between PD and HCs. Purple areas are cortical regions where FD was found to be higher in PD patients. Orange areas are subcortical regions where FD was found to be lower in PD patients (C) Hypergraph neural network-based classification of PD and HC test data (n=28) using the nine significant FD features achieved an area under the receiver operating characteristic (ROC) curve (AUC) of 0.88, and sensitivity and specificity of 0.86 and 0.79, respectively (D).

Validity of FD as a neurodegenerative biomarker was first investigated by comparing open-source, multi-center data from patients with PD (n = 70) and healthy controls (HCs; n = 70) and modelling FD against a proxy of disease severity in our large PD cohort (n = 231). A two-sided t-test with corrections for multiple comparisons revealed significant PD and HC group differences in FD (p < 0.01) for nine brain regions including: the left superior and medial frontal gyri, left supplementary motor area, left insular cortex, right inferior occipital area, bilateral paracentral lobules, right putamen, and right pallidum (Fig. 1AB). Cortical regions showed an increase in FD for patients with PD compared to HCs, while subcortical regions demonstrated lower FD in the patient group. The classification power of selected FD features was tested using a hypergraph neural network (HGNN) learning model which achieved an area under the receiver operating characteristic curve (AUC) of 0.88 with sensitivity and specificity of 0.86 and 0.79, respectively, for distinguishing patients with PD from HCs (Fig. 1BC). Regional FD showed significant correlations with disease severity in PD, inferred by patients’ preoperative motor scores while off PD medications (Table 1). Specifically, significant positive correlations were found for FD of the left superior frontal gyrus, left supplementary motor area, left gyrus rectus, left parahippocampal region, right lingual gyrus, left postcentral gyrus, left inferior parietal lobule, and right superior temporal gyrus.

### Selected features to predict outcomes.

For the main analysis, STN and GPi patients were combined, with the DBS target included as a covariate to maintain cohort size and to enhance generalizability and utility. To further explore target-specific effects, a subgroup analysis comparing STN and GPi patients was conducted, and detailed results of this analysis, including Ridge regression coefficients and statistical comparisons, are provided in the supplementary material. Feature selection via LASSO regression on the training dataset (n = 162) yielded 20 significant predictors including: the DBS target (STN vs. GPi), hemispheres treated (bilateral vs. unilateral), age at MRI, percent improvement in preoperative motor scores from the OFF- to ON-medication state, and FD of 16 brain regions localized to the frontal lobe, Rolandic cortex, cingulate cortex, amygdala, occipital lobe, paracentral lobule, and caudate nucleus (Fig. 2A). A LASSO shrinkage factor of 0.03 was selected based on optimal classification performance of corresponding predictive features selected and tested across a range of shrinkage factors (Fig. 2C). Beyond the DBS target, FD of the right paracentral lobule, left middle occipital gyrus, and right olfactory area were the three most important features with the highest normalized ridge regression coefficients (Fig. 2B). Mapping selected FD predictors onto a normalized T1-w image visually confirmed their spatial distribution throughout the brain (Fig. 2D). Individual differences in average FD could also be visually appreciated directly from segmented tissue boundaries on subject-specific T1-w images (Fig. 2E).

### Variance in outcomes explained by FD.

Ordinary least squares regression (OLS) was used to assess the variance in 6-month DBS outcomes (pre-to-post change in PD medications) explained by selected clinical and imaging features. Including selected FD features for the training dataset (n = 162) in a combined model with clinical features explained an additional 13.6% of the variance in patient outcomes (R^2^ = 0.388) compared to a model of clinical features alone (R^2^ = 0.252). An F-test comparing the two models yielded an F-statistic of 2.76 (p < 0.01), confirming that the inclusion of FD significantly improved the model fit.

### Classification performance of FD.

An HGNN classification learning task was performed to distinguish patients whose daily medication dose decreased versus increased (or did not change) following DBS. Optimal performance of the combined model was achieved using a model hyperparameter of k = 12, which defines how many nodes each hyperedge contains in a k-uniform hypergraph (Fig. 3A). Combining FD features with clinical features in a multi-feature hypergraph model trained and optimized on 196 patients and tested on 35, resulted in an AUC of 0.76 compared to 0.64 for a hypergraph model derived from clinical features alone (Fig. 3B). The inclusion of FD with clinical features increased the model’s sensitivity by 15 percentage points (from 0.54 to 0.69) and specificity by 22 percentage points (from 0.56 to 0.78), enhancing the overall accuracy in predicting changes in levodopa equivalent daily dose (LEDD) (Fig. 3C).

## Discussion

This retrospective study investigated the added value of T1-w measures of cortical and subcortical complexity in predicting PD outcomes with DBS. Few prior studies have used T1-w FD to discriminate patients with PD from HCs and reported mixed findings. Kubera et al. reported no significant differences in FD between 22 patients with PD and 18 HCs.^28^ Conversely, Li et al. investigated a larger sample of 60 patients and 56 HCs and identified significant reductions in FD for patients, particularly in cortical motor areas, indicating that FD could capture important cortical changes associated with PD progression.^29^ Our findings add to this small body of literature and echo other evidence of FD robustness across multiple-acquisitions^31^ by demonstrating that FD can effectively distinguish patients with PD from HCs using an unbiased classifier neural network model and open-source, multi-center data. The direction of FD group effects was region-specific, which we hypothesize could be reflective of distinct underlying processes at the subcortical and cortical levels. Lower FD values in PD subcortical areas such as the basal ganglia could indicate structural degeneration typical of PD progression.^32,33^ In comparison, higher FD values in cortical areas integral to motor, cognitive, and visuospatial functions affected in PD, may be due to the presence of inflammatory processes or cellular changes as hypothesized by one group which observed increased gray matter FD in patients with multiple sclerosis, compared to HCs.^34^ This phenomenon of abnormally increased cortical complexity has also been observed in the temporal cortices of patients with premanifest Huntington’s disease.^35^ Furthermore, a recent study in PD highlighted the importance of cortical compensatory processes in determining clinical disease severity, in conjunction with basal ganglia degeneration^36^, supporting a distinction between subcortical and cortical disease processes affecting structural integrity. The fact that our group level trends for cortical FD contradict observations by Li et al. despite a relatively similar sized cohort, suggests the presence of other sources of variability. For example, disease durations of our multi-site sub-cohorts were higher than the Li et al. cohort (3–4 years; range of approx. 1–10 years) at 9.4±4.9 years for our single-center data and 5.9±3.9 years for one of the two public datasets^37^ (unreported for the other dataset). Further investigation is required to validate and explain this contradiction; nonetheless, the significant predictive power of FD was sufficient here to still warrant it use as biomarker.

Our within-patient trends also reinforced the use of FD as a biologically meaningful biomarker. Regional FD correlated with preoperative motor symptom severity, measured using part III of the Movement Disorder Society Unified PD Rating Scale (MDS-UPDRS III) in the OFF-medication state.^38^ We used the virtual version of the MDS-UPDRS III scores which did not include tests of rigidity or postural instability because of limited in person visits during the pandemic. The FD of frontal cortical areas showed consistent positive correlations with motor severity, further indicating increased cortical complexity with greater disease severity. Indeed, FD of frontal areas were also significant in distinguishing patients from HCs, with patients showing increased cortical complexity within these structures on average. Collectively, these results suggest that the structural integrity of the frontal cortex plays a significant role in objectively characterizing PD. As our subsequent results showed, this structural integrity of the frontal cortex was also predictive of patient outcomes, in line with one prior study which demonstrated in 31 patients that frontal cortical thickness could predict clinical improvement following STN-DBS.^39^ This relationship between frontal cortical FD and outcomes may reflect broader network effects, given the frontal cortex’s connectivity with motor-related regions such as the precuneus and basal ganglia, including the pallidum. The precuneus, for instance, is interconnected with the premotor area and is involved in the complex coordination of motor, cognitive, and emotional functions. Similarly, the pallidum, which serves as a target for DBS in alleviating motor symptoms, may be indirectly affected by changes in cortical structure and function. These observations suggest that structural and network-level changes in the frontal cortex and its connected regions contribute to the pathophysiology of PD and influence clinical outcomes. It is also worth noting that correlations between FD and motor severity were slightly stronger in the ON- versus OFF-medication state, which could reflect a better approximation of the functional disease state mediated by structural integrity.

LASSO and ridge regression analysis revealed key features impacting DBS outcomes. The selected DBS target explained the most variance in patient outcomes, consistent with longstanding evidence that clinical response to STN and GPi DBS can differ^40,41^. The relative importance of this feature was expected to be high and is directly related to our chosen outcome metric which approximated response to DBS based on pre-to-post changes in dopamine medications. STN stimulation is known for effectively reducing motor symptoms and medication needs, while GPi stimulation, often chosen for patients with prominent dyskinesias as well as greater risk for cognitive decline, typically results in less medication reduction.^42^ While we combined STN and GPi groups in the main analysis to maintain generalizability, a target-specific subgroup analysis is included in the supplementary material. Other clinical features were also important, though less than regional FD features according to their ridge regression coefficient. A bilateral lead implantation was associated with more substantial symptom relief, consistent with clinical observations^43,44^. Age at the time of MRI was another impactful factor, with older patients potentially having different neural responses and adaptive capacities^45,46^. Finally, preoperative medication responsiveness, one of the gold standard clinical predictors of motor response to DBS, was also significantly related to outcomes. Although DBS acts through mechanisms distinct from pharmacological intervention,^47^ motor symptoms being levodopa responsive suggests that the network can be modified and that there are intact dopaminergic pathways. Our results highlight this value but also emphasize the added value of more objective predictors like FD.

Indeed, incorporating regional FD metrics into our model explained additional variance in DBS outcomes. Significant brain areas were distributed throughout the brain which is not surprising given the complex symptomatic nature of PD.^48^ For example, important FD predictors localized to the frontal lobe, amygdala, cingulate gyrus, insula, and temporal pole suggesting that DBS outcomes may depend not only on the structural integrity of motor circuitry but also areas involved in cognitive and emotional processing. This aligns with clinical evidence showing that patients with preserved cognitive function tend to have more favorable responses to DBS.^49^ FD of the paracentral lobule was one of the most informative features, with a ridge regression coefficient comparable to the chosen DBS target, reinforcing the relationship between structural integrity of motor areas and response to DBS.^25^ More specifically, given the role of the paracentral lobule in controlling lower extremity function, these results could potentially be indicative of a link between axial disability in PD and DBS outcomes. Indeed, research has shown that while STN-DBS may improve tremor and rigidity, its effects on axial symptoms, particularly gait and balance, are often less pronounced and can even worsen in some cases.^50^ Another interesting finding worth noting is the predictive value of FD of the olfactory lobe. Olfactory dysfunction, particularly hyposmia, is a prevalent non-motor symptom in PD, with incidence rates ranging between 62% and 98%.^51^ The double peak observed in the density plots for FD of the olfactory lobe (Fig. 2B) may reflect this variation in the severity of olfactory dysfunction among patients, reiterating the potential broader value of FD as a symptom-specific PD biomarker, though future dedicated studies are needed.

While variance analysis provides insights into factors driving DBS outcomes, practical application of imaging biomarkers requires predictive capability. Integrating FD metrics with clinical features in our HGNN model significantly improved predictive accuracy, with the combined model achieving higher performance metrics than the model trained on clinical features alone. To our knowledge, this is one of the first studies to employ a preoperative DBS prediction model in a large cohort with training, validation, and testing phases. We hypothesize that adding other modalities, such as functional and diffusion MRI and quantitative susceptibility mapping,^19–24^ will enhance predictive power and potentially identify clinical subgroups with distinct response patterns.

Clinically, DBS candidates are currently selected based on subjective predictors. A standardized approach to more objectively estimate the likelihood that an individual will respond well to the therapy has not yet been adopted. As the annual number of implantation surgeries continues to increase,^8,9^ there is a growing need for streamlined approaches to implementing DBS. T1-w MRI data are readily accessible in all clinical DBS settings, making our approach feasible for widespread adoption. A key implication of our findings is that, by using MRI-derived biomarkers like FD, clinicians may eventually be able to predict which patients will benefit most from DBS without the need for traditional ON-/OFF-medication motor testing. This could streamline the decision-making process and reduce the burden on patients undergoing DBS candidacy evaluations. It would also allow clinicians to better target DBS resources toward patients most likely to benefit and lead to more democratized access to advanced DBS planning tools and potentially broadening the availability of DBS for a larger and more diverse population of patient with PD.

Finally, we would like to acknowledge that this study has several limitations. Automated registration and segmentation tools, while efficient, do not match the accuracy of manual segmentation. Also, due to retrospective data constraints, we used the pre-to-post change in dopaminergic medications as the primary outcome metric, which may not accurately reflect true response to DBS by only partially capturing symptom improvements. For example, we did not consider the presence of dyskinesia or dopamine dependent depression which could influence neurologist strategies for modifying medication schedules following DBS. Many patients underwent preoperative virtual examination assessing on and off motor scores used in this study which excluded rigidity and postural stability testing so it may not totally accurately reflect patients’ true total motor scores. Clinical data variability due to different sites, scanners, and protocols over thirteen years also poses challenges by introducing differences in data quality. However, we also view this variability as a strength of the study since it reflects real-world logistics of acquiring clinical data, even at a single center, and enhances model generalizability. Given these limitations, we encourage validation and refinement of our findings using our published data (see methods section for access), other datasets with standardized imaging protocols, and more accurate measures of outcome such as change in on and off medication MDS-UPDRS III and neurocognitive assessments after DBS. Ongoing work from our group is focused on prospective collection of standardized outcome metrics and integration of additional MRI modalities, toward the development of a multimodal MRI-based decision support tool for DBS candidate selection.

In summary, this work demonstrates the potential of T1-w MRI-derived FD as a valuable biomarker for predicting PD from HC and PD DBS outcomes, offering a novel and practical approach to improving PD management. This research represents a significant step towards personalized medicine, enhancing the efficacy and accessibility of DBS therapy for a broader patient population.

## Methods

### Study population.

Preoperative MRI data were retrospectively collected from 231 patients (mean age 65 ± 9 years; 33% female) who underwent DBS surgery at the University of California, San Francisco (UCSF) between 2010 and 2023. With approval from UCSF’s institutional review board, all participants provided written informed consent to the use of their data for investigations of generic DBS outcomes. A comprehensive list of patient demographics and clinical parameters is provided in Table 2. A majority of patients (80%) received bilateral DBS implants, with 58% of all subjects implanted in the GPi and the remaining 42% in the STN. Subjects were separated into two outcome groups according to changes in their LEDD following approximately 6 months of active DBS with optimized stimulation parameters and medication schedules. The first group included patients with a ΔLEDD *less* than or equal to 0 who experienced no change in medication or who increased their daily medication dose, while the second group consisted of patients with a ΔLEDD *greater* than 0 who reduced their daily medication dose. A thorough description of this outcome metric is provided below.

### Outcome metric.

A prospective study is underway to collect standardized motor and cognitive outcome data for this cohort. Thus, this retrospective analysis inferred outcomes from documented changes in patients’ dopaminergic medications before versus after DBS surgery. Although ΔLEDD is not a direct measure of symptom response, it has previously been used as a surrogate measure to gauge clinical outcomes following DBS. Studies have shown that a reduction in LEDD generally correlates with positive motor outcomes, while an increase may indicate a suboptimal response to therapy^52,53^.

**Figure 4:**
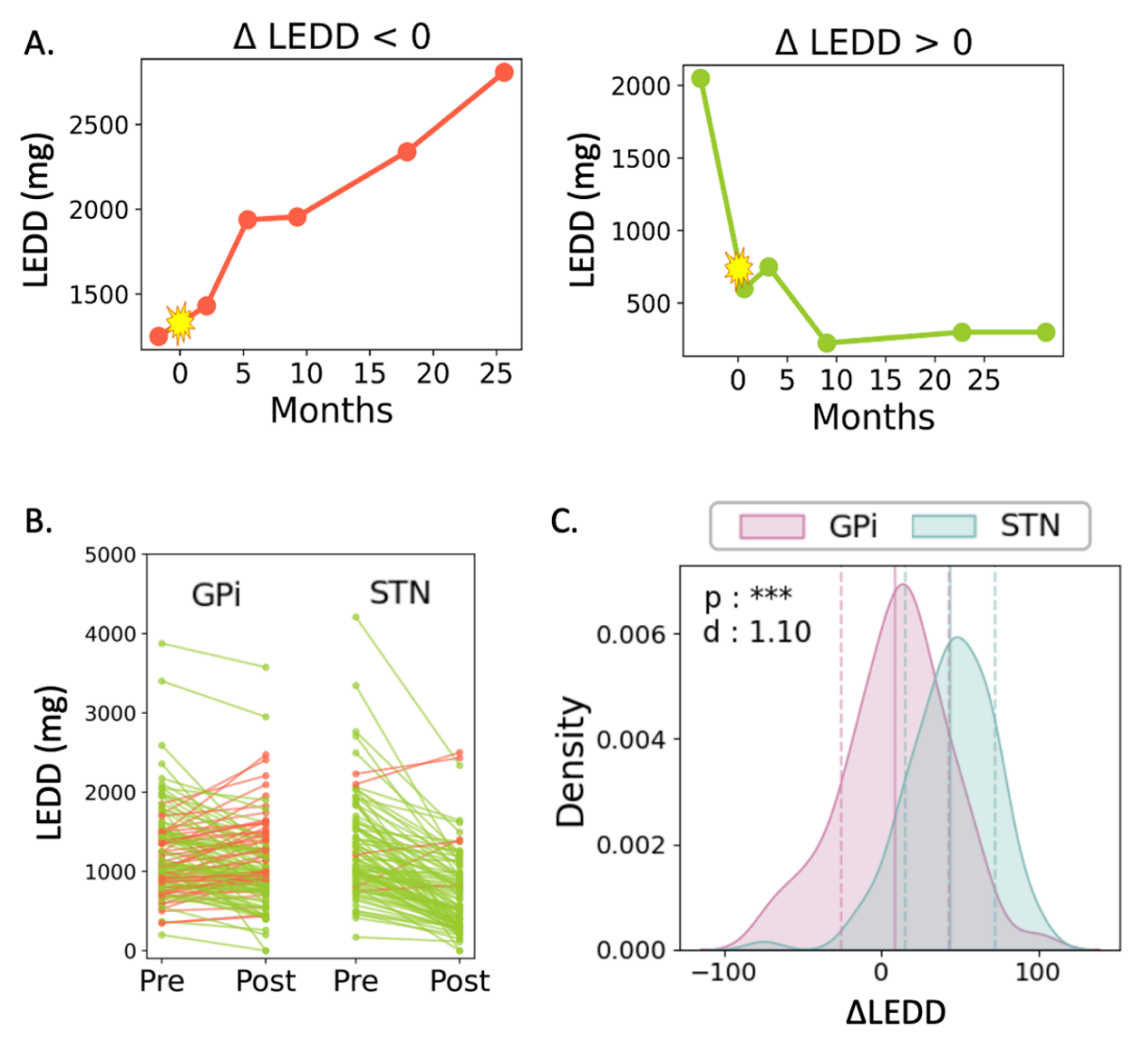
Individual and target-specific changes in dopaminergic medications following DBS: (A) Example LEDD trajectories over time for two patients: one with an increase in LEDD (ΔLEDD≤0) and one with a decrease (ΔLEDD>0). The zero-month mark in yellow indicates the date of DBS surgery. (B) Reductions in levodopa equivalent daily dose (LEDD) were observed (green lines) for most patients, irrespective of their DBS target. However, a subset of patients’ LEDD increased (red lines) following 6 months of DBS; this was mainly individuals implanted in the GPi. (C) Plotted distributions of LEDD change were significantly different for STN (n=96) and GPi (n=135) patient groups (t-test: p<0.001, Cohen’s d=1.10), further demonstrating more negative values (less reduction in LEDD) for GPi-implanted patients. The mean and standard deviation of each distribution are denoted as a vertical solid line and two dotted lines, respectively.

To calculate LEDD for each patient, a comprehensive review of medical records was conducted to extract medication schedules from all available patient visits up to 12 months before and 12 months after DBS surgery. The type, dosage, and intake frequency of each dopaminergic medication were meticulously documented by three experienced researchers (S.D., J.M., D.S.). Daily medication dosages were converted to their levodopa equivalent using literature-reported conversion factors,^54^ accounting for bioavailability and pharmacokinetics and enabling the comparison and aggregation of medication doses across different regimens. The pre-to-post change in LEDD was calculated as

$$\text{Δ}\;LEDD=100\%\times \frac{LEDD_{pre}-LEDD_{post}}{LEDD_{pre}}$$

with ${\text{LEDD}}_{\text{pre}}$ derived from the visit preceding DBS surgery and ${\text{LEDD}}_{\text{post}}$ from the visit nearest to 6 months of active DBS. This latter time point was selected based on data availability and the typical duration of DBS parameter optimization. Before incorporating ΔLEDD in our models, individual and target-specific trends were explored, reinforcing target effects and revealing two divergent patterns in LEDD over time that informed clinical feature selection and subject classification labels (Fig. 4).

### Image acquisition and FD quantification.

**Figure 5:**
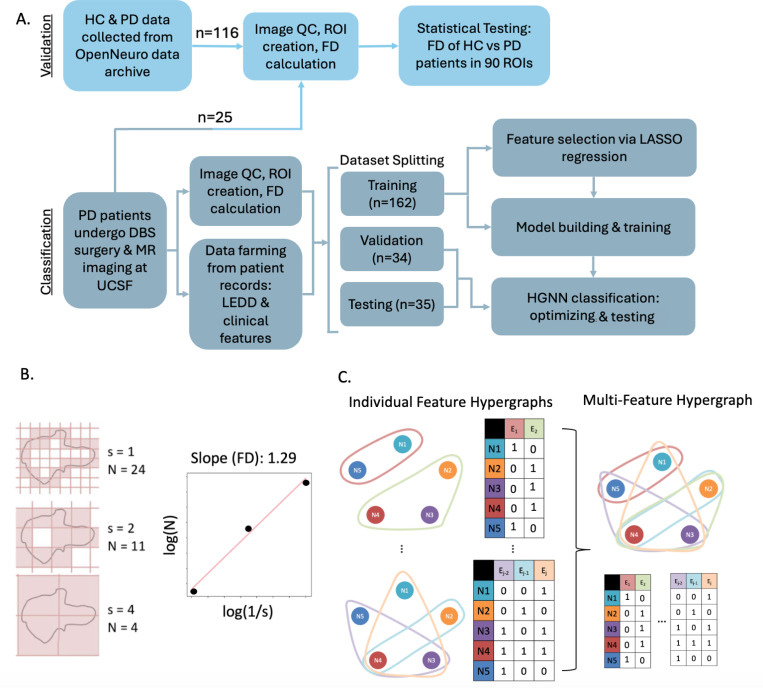
Overview of study methods: (A) Flow chart illustrating data sources and analysis steps for biomarker validation, variance analysis, and classification tasks. (B) Simple schematic of the box-counting method for calculating the fractal dimension of a surface. (C) Simple schematic of hypergraphs and their respective incidence matrices. Individual feature hypergraphs are concatenated horizontally to create a multi-feature hypergraph. ${\text{N}}_{\text{i}}=\text{node/subject}$, ${\text{E}}_{\text{j}}=\text{hyperedge}$.

A flowchart illustrating key methodological steps is provided in Fig. 5A. Preoperative imaging was performed between 2010 and 2023 on 3T MRI scanners of varying vendors and models with an 8- or 32- channel head coil. The T1-w imaging protocols had variable acquisition parameters reflecting real-world clinical settings, including repetition times = 680–1000ms, echo times = 3–4ms, field-of-view = 256×256mm, and voxel resolutions of 0.5×0.5×1mm or 1mm isotropic. All images underwent visual inspection to ensure adequate image quality, void of significant artifact affecting tissue and/or brain structure boundaries. The primary cohort (n = 231) includes only those subjects who had good quality data.

To quantify regional FD, whole-brain regions of interest (ROIs) were first mapped to patients’ native brain space. Affine and deformable transformations were performed using FMRIB Software Library (FSL)^55,56^ to register each patients’ brain images into the Montreal Neurological Institute standard space^57^. The inverse warp of the deformable transformation was then applied to the 90-ROI parcellation map from the Automated Anatomical Labeling (AAL-90) atlas^58^. In Python, FD was calculated for each patients’ ROIs warped to their native brain space. We used the box-counting method^59,60^ which involves iteratively measuring the number of non-overlapping boxes, N, of incrementing sizes, s, needed to cover the surface of each ROI. The process starts by setting the largest box size to the length of the smallest dimension of the 3D image. The algorithm then systematically reduces the box size in a logarithmic fashion, halving the size at each step. At each box size, the number of boxes required to cover the non-zero voxels is counted. Finally, the relationship between the box size and the number of boxes needed is plotted on a logarithmic scale, and the slope of this plot, obtained through linear regression, provides the fractal dimension. As shown in Fig. 5B, FD was taken as the slope of the log(N) versus log(1/s) relationship.

### Validation of FD as a PD biomarker.

Before evaluating the predictive value of regional FD, we sought to verify that the metric could discriminate individuals with PD from HCs and explain patient differences in disease severity. To reduce potential single-site bias and enhance study rigor, 70 HC datasets were sourced from three different studies and sites within OpenNeuro and open-source online collections^61–63^. Similarly, 70 PD datasets were selected from three different studies and sites,^37,64^ including 25 datasets randomly chosen from our primary UCSF cohort. To mitigate the effects of age on brain structure, HCs were age-matched to PD patients.

To ensure assumptions of normality were met for the residuals in regression analyses, we created quantile-quantile plots and performed the Anderson-Darling test, as detailed in the Supplementary Information. Following normality testing, two-sided t-tests were conducted to compare all 90 regional FD values across the PD and HC groups. False discovery rate correction for multiple comparisons was applied using the Benjamini-Hochberg method^65^. ROIs with an adjusted p-value < 0.05 were deemed statistically significant. The predictive power of FD in discerning between patients and HCs was determined by training and testing a classification HGNN model (see Data representation and hypergraph neural network classification subsection below).

Linear regression was thereafter used to evaluate the relationship between FD of 90 ROIs and disease severity in our primary cohort of 231 patients with PD. Raw total motor scores based on the preoperative MDS-UPDRS III exam were used as a proxy of disease severity, excluding rigidity and postural stability exam items (3.3 and 3.12) due to some patients undergoing virtual examination. The analysis was conducted using patients’ total motor scores evaluated in the OFF- and ON-medication states, and age at MRI and sex were modelled as covariates. In total, 180 regression models were run to cover all the brain regions and medication states, and the Benjamini-Hochberg method was used to correct for multiple comparisons. Correlations with a p-value < 0.05 were deemed significant and 95% confidence intervals were calculated for regression coefficients to assess the precision of each estimate.

### Feature selection.

A multi-step statistical approach was employed to investigate whether additional variance in DBS outcomes could be explained by T1-w regional FD features. In addition to the 90 regional FD features, clinical features considered included: age at the time of MRI, sex, DBS target (STN or GPi), hemispheres treated (unilateral or bilateral), and preoperative medication responsiveness quantified as the percent improvement in MDS-UPDRS III motor scores from the OFF- to ON-medication state (%Δ MDS-UPDRS III).

Given the high dimensionality of the dataset, we first implemented feature selection to prevent model overfitting and enhance interpretability. Feature selection was performed using only the training dataset (~ 70%), to ensure that the validation (~ 15%) and testing (~ 15%) data remained independent for unbiased evaluation. To complement the main analysis, we conducted a cross-validation analysis to evaluate feature selection consistency across the entire dataset (see Supplementary Information). All continuous features were normalized to have a mean of zero and standard deviation of one such that features with more extensive range could not disproportionately influence the results.

Lease Absolute Shrinkage and Selection Operator (LASSO) regression was applied due to its superiority for feature selection within high-dimension datasets ^66^. LASSO penalizes the absolute size of the regression coefficients, effectively shrinking some of them to zero, which simultaneously achieves feature selection and regularization. To select the optimal shrinkage factor (α), we employed 5-fold cross-validation, selecting the α that maximized the ROC AUC of the classification model. Selected features provided a refined set of predictors for constructing our hypergraphs for variance analysis and classification learning tasks.

Ridge regression was thereafter performed to incorporate the complete set of LASSO-selected features while addressing multicollinearity and enhancing model stability^67^. Ridge regression, which penalizes the square of the regression coefficients, was chosen for its ability to handle multicollinearity by shrinking the coefficients of correlated predictors towards each other. This method yielded coefficients representing the relative importance of each selected feature in explaining variance in DBS outcomes. Normalized coefficients provided weights for our HGNN model described below as a pseudo representation of feature importance in the combined multi-feature hypergraph structure.

### Variance analysis.

We sought to quantify the additional variance in LEDD outcomes explained by FD features when combined with predictive clinical features. To achieve this, we performed a variance analysis comparing two OLS regression models. The first model included only clinical features as predictors including: the DBS target (STN or GPi), hemisphere treated (unilateral vs. bilateral), age at the time of MRI, sex, and the preoperative change in MDS-UPDRS scores from the OFF- to ON-medication state. The second model included all predictors from the first clinical model plus selected FD features derived from preoperative T1-w MRI scans.

Both models were fit using the OLS method, and respective R-squared values were compared to assess the proportion of variance explained by each model. To determine whether the inclusion of FD significantly improved the model’s explanatory power, we performed an F-test for nested models. The F-test compared the fit of the two models by testing the null hypothesis that adding imaging features would not improve the model significantly over the clinical features alone. Specifically, the F-statistic was calculated as

$$F=\frac{\left(RSS_{1}-RSS_{2}\right)/\left(df_{2}-df_{1}\right)}{RSS_{2}/df_{2}}$$

where ${\text{RSS}}_{\text{i}}$ and ${\text{df}}_{\text{i}}$ are the individual models’ residual sum of squares and degrees of freedom, respectively. The resulting F-statistic was used to calculate the p-value indicating whether additional variance explained by the imaging features was statistically significant.

### Data representation and hypergraph neural network classification.

We constructed hypergraph structures based on the LASSO-selected features to capture the complex relationships between features. Each feature formed a hypergraph, with nodes representing subjects and hyperedges representing similarity or categorical grouping within the feature space.

For categorical features such as DBS target (STN vs. GPi), hemispheres implanted (unilateral vs. bilateral), and sex, hyperedges were formed by grouping subjects based on their category. For continuous variables such as preoperative motor scores and regional FD, we created k-uniform hypergraphs with k varying from 5 to 13 to assess the impact of different connectivity scales. This range of k-values was chosen empirically to ensure stable results to the point of plateau. The k-uniform hypergraphs were constructed by connecting each node to its k-1 nearest neighbors based on feature similarity. The edges of all hypergraphs were weighted using the normalized coefficients from ridge regression that reflect relative feature importance. The hypergraphs of individual features were concatenated horizontally, as shown in Fig. 5C, to create multi-feature hypergraphs. Two hypergraphs were constructed, one with only clinical features and one that contained clinical features and FD features. Both hypergraphs were used to independently train the classification model to test if the addition of FD improved predictive accuracy and by how much.

The construction of hypergraphs was implemented using the DeepHyperGraph PyTorch package^68^, which provides efficient tools for HGNN construction and training. HGNNs are designed to process data structured as hypergraphs. In the HGNN framework, the key components include hyperedge convolution layers, node-edge-node transformations, and spectral convolution operations. Spectral convolution is at the core of the HGNN architecture, where signals defined on hypergraphs are convolved with learnable filters to extract features that capture high-order correlations inherent in high-dimensional data.

Our HGNN model was comprised of multiple layers, each responsible for transforming node features through convolutional operations. Initially, node features were processed by learnable filter matrices, resulting in feature representations that captured local and global structural information within the hypergraph. Hyperedge features were then derived through aggregation mechanisms that leveraged the hyperedge connectivity information encoded in the multi-feature hypergraph incidence matrix. This node-edge-node transformation process allowed our HGNN model to effectively capture and propagate information across different scales of connectivity within the hypergraph, facilitating the extraction of discriminative features for downstream classification tasks^69^. The final convolutional layer performed classification to predict the likelihood of each subject’s ΔLEDD being less than or equal to 0, or greater than 0. The model was trained using a supervised learning approach, with cross-entropy loss as the objective function.

Model hyperparameters were optimized using the validation dataset, and performance was monitored at each epoch using the validation subset to identify the best-performing model. The final model performance was evaluated on the test subset. The ROC AUC was calculated for the clinical features hypergraph model and the combined model with both clinical and FD features to assess differences in classification accuracy and overall model performance.

## Supplementary Material

Supplement 1

## Figures and Tables

**Table 1:** Relationship between fractal dimension and preoperative motor scores.

Region	p	R	95% CI
Frontal Sup Orb L	*	0.12	[0.009, 0.248]
Supp Motor Area L	*	0.13	[0.001, 0.258]
Rectus L	*	0.12	[−0.003, 0.249]
Parahippocampal L	*	0.13	[0.002, 0.259]
Lingual R	**	0.21	[0.078, 0.328]
Postcentral L	*	0.13	[0.000, 0.255]
Parietal Inf L	**	0.16	[0.032, 0.286]
Temporal Sup R	*	0.13	[0.005, 0.252]

* p < 0.05,

** p < 0.01

**Table 2:** Demographics and treatment parameters of primary PD cohort.

Clinical Feature	Total	ΔLEDD≤0	ΔLEDD>0
Subjects (n)	231	60	171
Male (n)	155	40	115
Female (n)	76	20	56
Mean Age (years)	65 ± 9	66 ± 7	64 ± 10
STN Target (n)	96	6	90
GPi Target (n)	135	54	81
Unilateral (n)	44	15	29
Bilateral (n)	187	45	142
Mean Motor Improvement (%)^a^	53 ± 16	54 ± 15	52 ± 17
Mean ΔLEDD (%)^b^	23 ± 36	−22 ± 22	39 ± 25

a based on preoperative MDS-UPDRS III exam on- and off-medications

b based on patients’ dosage regimen before versus after approx. 6-months of DBS

## Data Availability

In line with promoting transparency and reproducibility in scientific research, we have made example processing and analysis data, including templates for the MDS-UPDRS and LEDD calculators, publicly available on GitHub (Radiology-Morrison-lab-UCSF/T1w_FractalDimension). The repository also includes a de-identified example T1-weighted MRI scan and corresponding fractal dimension (FD) values for 90 regions of interest (ROIs) from the AAL atlas, enabling users to reproduce at least one FD calculation for a single subject. The repository is actively being updated to include code for image processing workflow, fractal dimension calculation, hypergraph classification for neuroimaging datasets, and statistical analysis pipelines. The full dataset, including raw MRI images and comprehensive de-identified clinical features, will be made publicly available on OpenNeuro in a future publication.
